# Effects of Ascorbic Acid on Apoptosis, Metabolism, and Muscle Quality in Ammonia-Stressed Rainbow Trout (*Oncorhynchus mykiss*)

**DOI:** 10.3390/foods15132316

**Published:** 2026-06-30

**Authors:** Siliang Yuan, Yiwen Wu, Yuxuan Pi, Chenxin Wang, Guangquan Xiong, Wenjin Wu, Liu Shi, Tao Yin, Hao Du, Lan Wang, Sheng Chen

**Affiliations:** 1 Yangtze River Fisheries Research Institute, Chinese Academy of Fishery Sciences, Wuhan 430223, China; 2 Key Laboratory of Agricultural Products Cold Chain Logistics, Ministry of Agriculture and Rural Affairs, Hubei Key Laboratory of Characteristic Resources and Utilization, Institute of Agro-Products Processing and Nuclear Agricultural Technology, Hubei Academy of Agricultural Sciences, Wuhan 430064, China; 3 College of Food Science and Technology, Huazhong Agricultural University, Wuhan 430070, China; 4 School of Bioengineering and Food Science, Hubei University of Technology, Wuhan 430068, China; 5 College of Biological and Food Engineering, Hubei Minzu University, Enshi 445000, China

**Keywords:** ammonia stress, *Oncorhynchus mykiss*, ascorbic acid, apoptosis, muscle quality

## Abstract

The present study aimed to evaluate the role of ascorbic acid in alleviating ammonia-induced muscle quality deterioration and to clarify its regulatory effects on apoptosis, texture, and flavor-related metabolites in rainbow trout (*Oncorhynchus mykiss*). The results demonstrated that ascorbic acid alleviated ammonia stress-induced inflammatory and apoptotic damage by regulating toll like receptor 5 (*TLR5*), myeloid differentiation primary response 88 (*MyD88*), and nuclear factor kappa-light-chain-enhancer of activated B cells (*NF-κB*) expression, thereby contributing to the restoration of myofibrillar integrity, reduced extracellular gaps, and increased shear force from 14.18 N to 18.26 N (*p* < 0.05). Ascorbic acid modulated ammonia handling and ion-exchange responses by upregulating glutamine synthetase (*GS*) expression from approximately 2.3-fold to 6.7-fold and increasing ornithine and citrulline accumulation. Alterations in tricarboxylic acid cycle-related metabolites further suggested that energy metabolism may be involved in the physiological adaptation to ammonia stress. Meanwhile, the ascorbic acid reduced the accumulation of key off-flavor compounds (1-octene-3-alcohol and (E)-2-nonenal), attenuating the earthy–moldy and fishy flavor. This research proposes a potential strategy to improve muscle quality in live transportation.

## 1. Introduction

In aquaculture systems, ammonia accumulation deteriorates water quality, which is produced from unconsumed bait and excretion of aquatic animals. The entry of molecular ammonia (highly toxic to aquatic animals) into fish occurs primarily via passive diffusion across the gill epithelium. Ammonium ions enter into gill cells through the Na/H exchanger (*NHE*) and enter into the gill epithelia cells via the Na^+^/K^+^-ATPase, finally being transported to the internal environment through the blood [1]. Once a fish is subjected to excessive ammonia, the stress makes the antioxidant system promptly respond, and might induce an inflammatory reaction, apoptosis, and finally result in tissue damage. It has been revealed that toll like receptors 5 (*TLR5*) inhibited oxidative stress and inflammation responses via inactivating nuclear factor kappa-light-chain-enhancer of activated B cells (*NF-κB*) and mitogen-activated protein kinase (*MAPK*) signaling pathways to reduce ammonia-induced inflammation, apoptosis, and tissue damage [2]. Meanwhile, apoptosis induces the destruction of muscle fibers and further affects muscle texture [3]. Hence, it is necessary to conduct a systematic investigation of the inflammatory response, apoptosis, and changes in muscle texture in response to ammonia stress.

When endogenous ammonia concentration accumulates to an excessive level in fish, they regulate energy metabolism and promote ammonia excretion to mitigate ammonia toxicity. Although it is a crucial strategy to improve tolerance to ammonia for the species to cope with ammonia stress, specific ammonia transporters and diverse detoxification mechanisms are necessary for the endogenous self-regulation of fish [4]. There are two pathways for ammonia detoxification: one involves the combination of ammonia with glutamate to produce non-toxic glutamine, and the other converts ammonia into urea through the ornithine–urea cycle pathway, which has been reported in rainbow trout and yellow catfish [5]. However, an increase in energy expenditure is driven by compensatory metabolic adjustments [6]. Energy-related metabolites including glucose, adenosine triphosphate (ATP), and inosine monophosphate (IMP) play vital roles in flavor formation and postmortem quality deterioration of fish muscle. However, limited information is currently available regarding the changes in specific flavor components of fish under ammonia stress, especially from the perspective of the coordinated regulation of ammonia detoxification and energy metabolism.

Rainbow trout (*Oncorhynchus mykiss*) is an economically valuable and delicious freshwater fish widely cultivated worldwide, and its global production reached 1,150,496 tons in 2023 [7]. It is highly sensitive to environmental stressors such as ammonia, temperature, and stocking density. Previous studies have demonstrated that dietary supplementation with glutamate [8], γ-aminobutyric acid [9], and vitamin A [10] can effectively alleviate environmental stress in rainbow trout. However, relevant research on nutrient supplementation during ammonia stress exposure remains scarce. Furthermore, the underlying correlations among ammonia transport, metabolism, and subsequent changes in filet texture and flavor, as well as targeted mitigation strategies, have not yet been fully elucidated.

Based on our previous studies, the threshold of the rainbow trout antioxidant system was set at a total ammonia concentration of 30 mg/L for 9 h [11,12,13]. Although ascorbic acid has been widely investigated as an effective strategy to alleviate environmental stress in fish, most previous studies have mainly focused on growth performance, antioxidant defense, immune regulation, inflammatory response, and tissue protection. However, less attention has been paid to how it affects the edible muscle quality of fish exposed to ammonia stress, especially from the perspective of apoptosis and metabolic remodeling. Therefore, this study aimed to evaluate the role of ascorbic acid in alleviating 9 h ammonia-induced deterioration of muscle quality and to clarify its regulatory effects on apoptosis, texture, and flavor-related metabolites in rainbow trout. The findings will provide a theoretical basis for establishing targeted environmental stress regulation strategies in live transportation, so as to improve fish health status and optimize muscle texture and flavor quality.

## 2. Methods and Materials

### 2.1. Materials

Rainbow trout (*Oncorhynchus mykiss*) with a weight of 1.5 ± 0.2 kg were acquired in Hanchuan, China. The water quality parameters of the aquaculture facility were recorded as follows: water temperature 16.1 °C, dissolved oxygen 11.2 mg/L, pH 7.07, and total ammonia nitrogen 1.41 mg/L. The pH, temperature, and dissolved oxygen were monitored using a Hassey water quality meter (HQ40D, HACH China Headquarters, Shanghai, China) and regulated using an ice and oxygenation pump. The total ammonia concentration in water was regulated by NH_4_Cl (analytically pure, ≥99.5%, China National Medicines Corporation Ltd., Beijing, China) and detected through the Nessler reagent method. Rainbow trout were randomly divided into three groups, with nine fish per group and a water-to-fish ratio (*v*/*w*) of 9:1.

### 2.2. Exposure Assay and Sample Collection

Based on our previous studies, the threshold of the rainbow trout antioxidant system was set at a total ammonia concentration of 30 mg/L for 9 h [11,12,13]. The experiment was set up with 3 groups: the control group (no NH_4_Cl addition), the stress group (exposed to 30 mg/L total ammonia for 9 h), and the relief group (exposed to 30 mg/L total ammonia for 9 h with 30 mg/L ascorbic acid). At the end of exposure, fish were anesthetized with 50 mg/L tricaine methane sulfonate (MS-222, Shanghai Macklin Biochemical Co., Ltd., Shanghai, China). Blood was then collected from the tail vein, and dorsal muscle was taken to determine shear force and water-holding capacity within an hour. And parts of the gill, kidney, and muscle were fixed with 4% paraformaldehyde for hematoxylin–eosin staining to observe tissue structure. The rest of the tissues were immediately frozen in liquid nitrogen, then stored at −80 °C before analysis.

### 2.3. Ammonia Transport

#### 2.3.1. Ammonia Concentrations in Tissues

The ammonia concentrations in blood, gill, kidney, and muscle were tested using the kit (A086-1-1, Nanjing Jiancheng Bioengineering Institute, Nanjing, China). *n* = 9 biological replicates.

#### 2.3.2. Relative Expression Level of NHE

The primer sequences of the genes are presented in Supplementary Material S1. Total RNA was extracted from tissue using trizol reagent (R701, Vazyme Biotech Co., Ltd., Nanjing, China). First-strand cDNA synthesis was acquired using a commercial kit (R323) (Vazyme Biotech Co., Ltd., Nanjing, China). cDNA purity was tested with the NanoDrop 2000 (Thermo Fisher Scientific, Waltham, MA, USA). The 10 μL reaction system consisted of 5 μL of master Mix (Vazyme, Nanjing, China), 0.5 μL of each primer mix (0.2 μmol/L), 1 μL of cDNA, and 3.5 μL of nuclease-free ddH_2_O added in a PIKOREAL 96 PCR System (Thermo Fisher Scientific, USA). The PCR thermal cycling program was as follows: 95 °C for 5 min, followed by 40 cycles of 95 °C for 10 s and 60 °C for 30 s. The relative expression changes were calculated using the 2^−△△Ct^ method. The gene expression levels were normalized to the reference gene *β-actin*. *n* = 6 biological replicates.

#### 2.3.3. Na^+^-K^+^-ATPase Activity

The Na^+^-K^+^-ATPase activity was detected using a kit (A016-1-1, Nanjing Jiancheng Bioengineering Institute, Nanjing, China). *n* = 6 biological replicates.

### 2.4. Tissue Damage

#### 2.4.1. Hematoxylin–Eosin Staining in Gill, Kidney, and Muscle

The gill, kidney, and muscle were fixed in 4% paraformaldehyde, dehydrated, embedded, and then cut at 5 μm thickness and stained with hematoxylin–eosin. The morphology and distribution of these tissues were examined under light microscopy (Eclipse Ci, Nikon Corporation, Tokyo, Japan). *n* = 3 biological replicates.

The hepatic histological changes were further quantitatively evaluated according to a protocol proposed by Corbett et al. (2015) [14]. A severity score value from 0 to 6 was assigned for the degree and extent of each alteration: 0—unchanged, (1 or 2)—mild, (3 or 4)—moderate, (5 or 6)—severe.

#### 2.4.2. The Relative Expression Level of Inflammation-Related Genes

qRT-PCR was used to detect the expression level of inflammation-related genes (*TLR 5*, *MyD88*, *NF-κB*, tumor necrosis factor (*TNF*), interleukin-6 (*IL-6*), complement component 4 (*C4*), and caspase-1 (*casp-1*). The primer sequences of the genes are presented in Supplementary Material S1. qRT-PCR was performed using the same protocol in Section 2.3.2. *n* = 6 biological replicates.

#### 2.4.3. TUNEL Immunohistochemistry

The number of apoptotic cells in gill, kidney, and muscle tissues was detected by the DeadEnd™ Fluorometric TUNEL System. The paraffin-embedded gill, kidney, and muscle tissues were cut at 3 μm thickness, then deparaffinized in xylene, absolute ethanol, and distilled water, and incubated with Proteinase K repair solution, and finally stained with TUNEL and DAPI. Green fluorescence was employed to identify apoptotic cells, while DAPI staining served as a blue background. Apoptotic index (%) = number of TUNEL-positive cells/total number of DAPI-stained nuclei × 100. *n* = 3 biological replicates.

### 2.5. Metabolites Related to Ammonia Detoxification

Ornithine, citrulline, arginine, aspartic acid, and glutamic acid was determined according to Wu et al. [12]. *n* = 4 biological replicates.

qRT-PCR was used to detect the expression level of glutamine synthetase genes (*GS*). The primer sequences of the genes are presented in Supplementary Material S1. qRT-PCR was performed using the protocol in Section 2.3.2. *n* = 6 biological replicates.

The content of α-ketoglutaric acid, cis-aconitate, succinic acid, fumaric acid, and malic acid was based on Dong et al. [15] with some modifications (detail in Supplementary Material S2). *n* = 4 biological replicates.

### 2.6. Energy Metabolism

The content of cortisol, glycogen, and pyruvic acid was determined using commercial kits (Nanjing Jiancheng Bioengineering Institute, Nanjing, China) H094-1-2, A043-1-1, and A081-1-1, respectively. *n* = 6 biological replicates.

Adenosine triphosphate (ATP), adenosine diphosphate (ADP), adenosine monophosphate (AMP), inosine monophosphate (IMP), hypoxanthine nucleoside (HxR), and hypoxanthine (Hx) were separated by HPLC (Ultimate 3000, Thermo Fisher Scientific, Dreieich, Germany) in 1.0 mL/min and 0.05 mol/L phosphate buffer (NaH_2_PO_4_ and Na_2_HPO_4_, pH 6.8) and detected at 254 nm. Energy charge (EC) was calculated by the equation from Zhang et al. [16]:

Energy Charge = (ATP + 0.5ADP)/(ATP + ADP + AMP). *n* = 4 biological replicates.

### 2.7. Volatile Compound Determination

The volatile compounds were separated and detected in gas chromatography (8890, Agilent Technologies Co., Ltd., Palo Alto, CA, USA) equipped with mass selective detector (7000D, Agilent Technologies Co., Ltd., Palo Alto, CA, USA) (detail in Supplementary Material S3). *n* = 4 biological replicates.

### 2.8. Muscle Quality

#### 2.8.1. The mRNA Expression Level of Genes Related to the Muscle Fiber Differentiation

The primer sequences of the genes (growth hormone receptor (*GHR*), insulin-like growth factor (*IGF*), protein kinase B (*Akt*), mechanistic target of rapamycin (*mTOR*), *myogenic differentiation 1* (*MyoD*), myogenin (*MyoG*), and calpain-2 (*Capn2*) are presented in Supplementary Material S1. qRT-PCR was performed using the protocol in Section 2.3.2. *n* = 6 biological replicates.

#### 2.8.2. Shear Force and Water-Holding Capacity

The muscle was cut into similar sizes (rectangular prism, 1 cm × 1 cm × 4 cm), then subjected to the digital tenderness meter (C-LM3B, Northeast Agricultural University, Haerbing, China) until the samples were cut down to get the value of the shear force. *n* = 9 biological replicates.

The muscle was trimmed into a cube (2 g) and wrapped in a piece of gauze (4 × 4 cm), then wrapped in filter paper, and finally centrifuged at 6000 rpm for 10 min at 4 °C. The water-holding capacity was the percentage of the muscle mass after centrifugation to initial muscle mass. *n* = 9 biological replicates.

### 2.9. Statistics

All data were expressed as mean ± standard error. When the assumptions of normality and homogeneity of variance were satisfied, differences among groups were analyzed by one-way analysis of variance (ANOVA), followed by Tukey’s honestly significant difference (HSD) test for multiple comparisons. Statistical significance was set at *p* < 0.05. Pearson’s correlation analysis was performed to assess associations among selected variables.

## 3. Result

### 3.1. The Influence of Ascorbic Acid Addition on the Immune System Under Ammonia Stress

**Figure 1:**
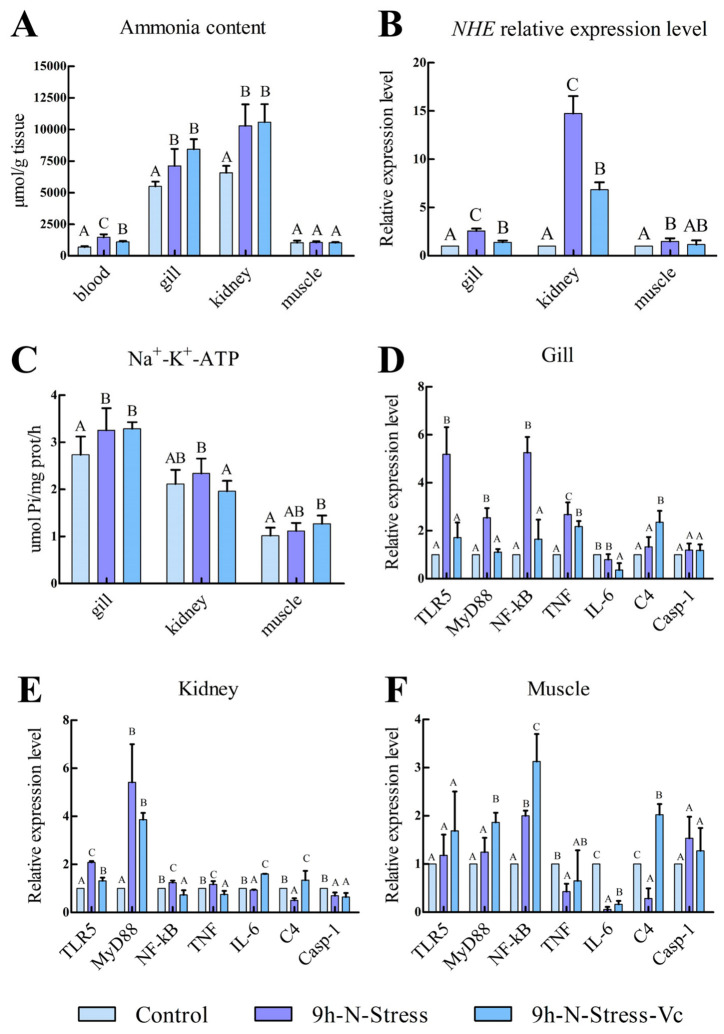
Effects of ascorbic acid addition on alleviating ammonia stress in ion exchange and the inflammatory response in *Oncorhynchus mykiss*. (**A**) The ammonia concentrations in the blood, gill, kidney, and muscle. (**B**) The Na^+^/H^+^ Exchanger (*NHE*) relative expression level in gill, kidney, and muscle. (**C**) The Na^+^-K^+^-ATPase in the gill, kidney, and muscle; the relative expression of genes [toll-like receptor 5 (*TLR5*), myeloid differentiation primary response 88 (*MyD88*), nuclear factor kappa-light-chain-enhancer of activated B cells (*NF-κB*), tumor necrosis factor (*TNE*), Interleukin-6 (*IL-6*), Complement Component 4 (*C4*), and caspase-1 (*Casp-1*)] related to the inflammatory response in gill (**D**), kidney (**E**), and muscle (**F**). Different uppercase letters mean significant differences (*p* < 0.05).

The ammonia levels in the blood, gill, kidney, and muscle are shown in Figure 1A. The levels in the gill and kidney were higher than those in the blood and muscle; the ammonia exposure led to a significant increase (*p* < 0.05) in the blood, gill, and kidney, but the ammonia levels in recovering fish did not differ significantly from those in the ammonia-exposed group (*p* > 0.05). The *NHE* mRNA expression in the gill, kidney, and muscle was observably upregulated by ammonia stress relative to the unchallenged condition (*p* < 0.05), and it was downregulated after recovery (Figure 1B). The Na^+^-K^+^-ATPase activity was higher in the ammonia-exposed group than that in control. Notably, in the gill and muscle, the highest activity was recorded under recovery, whereas in the kidney, recovery led to the lowest activity among all conditions (Figure 1C).

As seen in Figure 1D, the gill *TLR5*, *MyD88*, *NF-κB*, and *TNF* expression was significantly upregulated after ammonia exposure compared to the unchallenged condition (*p* < 0.05), while the *IL-6*, *C4*, and *casp-1* mRNA expression presented little difference between the two groups (*p* > 0.05). The gill *TLR5*, *MyD88*, *NF-κB*, *TNF*, and *IL-6* mRNA expression levels were downregulated in the relief group (*p* < 0.05), while *C4* mRNA expression reached significantly higher levels (*p* < 0.05), and the *Casp-1* mRNA expression showed no significant difference (*p* > 0.05). As for the kidney, the ammonia stress led to a tremendously higher mRNA expression level of *TNF* and a lower mRNA expression level of *IL-6* and *Casp-1* in comparison with the control (*p* < 0.05), but these gene expression levels appeared reduced, except that *IL-6* notably increased in the relief treatment compared with the ammonia stress (Figure 1E). In muscle, only *NF-κB* mRNA expression severely increased (*p* < 0.05), and *TNF*, *IL-6*, and *C4* showed the opposite tendency between the stress and control groups (*p* < 0.05). The relief group presented extremely increased expression in *MyD88*, *NF-ΚB*, *IL-6*, and *C4* (*p* < 0.05), and was slightly increased in *TLR5* and *TNF* in comparison with the ammonia stress (Figure 1F).

**Figure 2:**
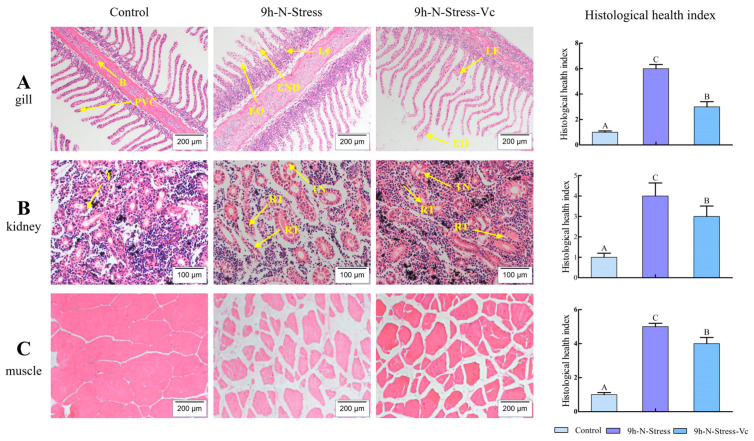
Effects of ascorbic acid addition on alleviating ammonia stress in tissue damage in *Oncorhynchus mykiss*. Hematoxylin–eosin staining and histological health indexes in gill (**A**, at 10×), kidney (**B**, at 20×), and muscle (**C**, at 10×). Gill: B: blood cells; PVC: respiratory epithelial cells; EO: vacuolation of epithelial cells; END: necrosis and shedding of epithelial cells; LF: layered fusion. Kidney: T: renal tubules; RT: shrunken lumen of renal tubules; TN: renal tubular necrosis. Different uppercase letters mean significant differences (*p* < 0.05).

Histopathological observations of gill, kidney, and muscle are presented in Figure 2A–C. In the control, gill structures remained intact with arranged lamellae and no notable pathological alterations. The gill filaments showed atrophy, with blurred outlines, and the epithelial cells showed disordered arrangement, with cellular edema and necrosis present after stress. In the relief treatment, gill filaments displayed layered fusion with no blurred outlines and atrophy, along with only mild cellular edema and vacuolation of the epithelial cells (Figure 2A). The kidney tissue showed a normal structure with abundant renal tubules, loose cytoplasm, and unobstructed lumen in the control. As for the ammonia stress, degeneration and even necrosis of the tubular epithelial cells and irregular cavities were observed, but less degeneration or necrosis of tubular epithelial cells was shown with ascorbic acid addition (Figure 2B). Ammonia exposure induced histological structural damage in muscle tissue, while the addition of ascorbic acid markedly alleviated such structural impairment. In the stress group, the myocyte gaps gradually became larger and more irregular and heterogeneous (Figure 2C).

**Figure 3:**
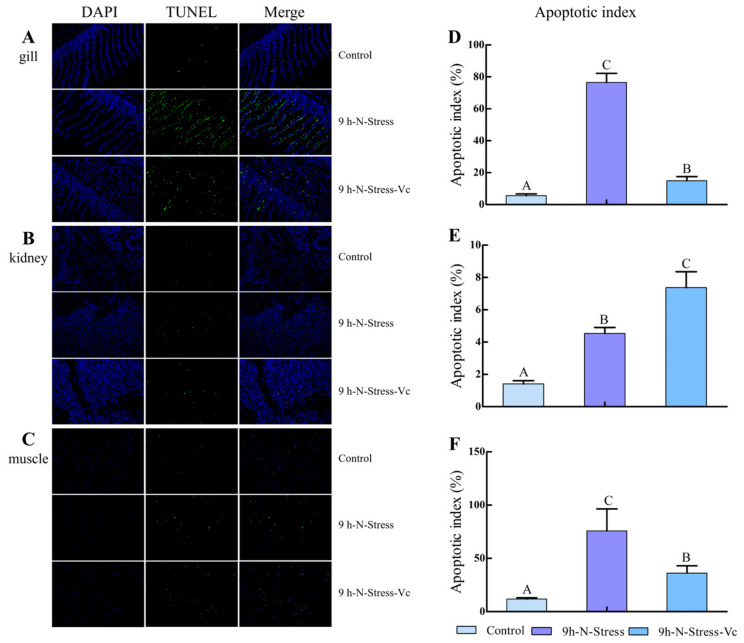
Effects of ascorbic acid addition on alleviating ammonia stress in apoptosis in *Oncorhynchus mykiss*. The TUNEL immunohistochemistry in gill (**A**), kidney (**B**), and muscle (**C**). The apoptotic index of gill (**D**), kidney (**E**), and muscle (**F**). Green fluorescence was employed to identify apoptotic cells, while DAPI staining served as a blue background. Different uppercase letters mean significant differences (*p* < 0.05).

The gill TUNEL analysis results (Figure 3A) showed only a small number of apoptotic cells (cells in green fluorescence) in the control, a dramatic growth in number with stress, and a sharp drop after relief in comparison with the stress. As for the kidney (Figure 3B) and muscle (Figure 3C), a notable increase in the occurrence of apoptotic cell death was observed in the stress and relief sets in comparison with the control.

### 3.2. The Influence of Ascorbic Acid Addition on Ammonia Metabolism and Energy Metabolism Under Ammonia Stress

**Figure 4:**
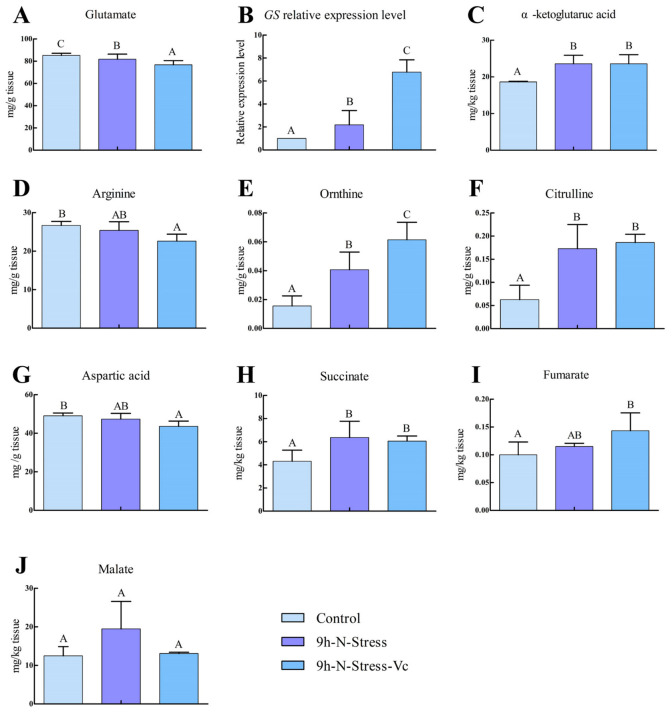
Effects of ascorbic acid addition on alleviating ammonia stress in ammonia metabolism in *Oncorhynchus mykiss*. (**A**): Glutamate; (**B**): glutamine synthetase (*GS*) relative expression level; (**C**): α-ketoglutaric acid; (**D**): arginine; (**E**): ornithine; (**F**): citrulline; (**G**): aspartic acid; (**H**): succinate; (**I**): fumarate; (**J**): malate. Different uppercase letters mean significant differences (*p* < 0.05).

As presented in Figure 4, the lowest α-ketoglutaric acid content and the highest glutamic acid content were both observed in the control. However, glutamic acid was lower with ascorbic acid addition (*p* < 0.05). The mRNA expression levels of *GS* were upregulated under stress, and reached the highest peak after ascorbic acid recovery (*p* < 0.05). The ornithine level was ascended in the stress group and reached its peak in the relief group (*p* < 0.05); the trend in the arginine and aspartic acid content was opposite to this. And a striking difference in citrulline was noted in the stress and relief groups compared with the control. The cis-aconitate content showed a marked increase after stress, and further rose with ascorbic acid addition (*p* < 0.05). As for the succinic acid, there was a remarkable increase when the fish were exposed to the ammonia stress (*p* < 0.05). The fumaric acid concentration in the relief group was significantly increased (*p* < 0.05), and there was no notable difference between the control and stress groups. The maximum value of malic acid was observed under ammonia.

**Figure 5:**
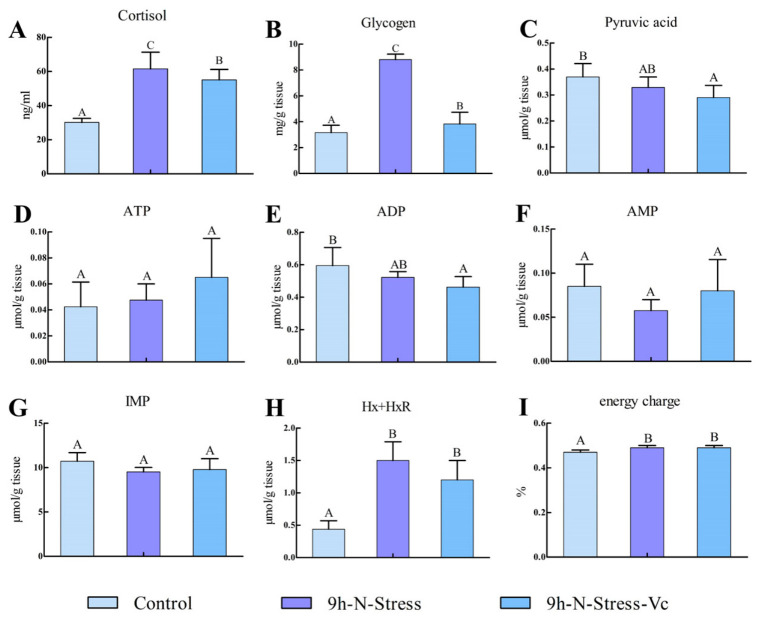
Effects of ascorbic acid addition on alleviating ammonia stress in energy metabolism in *Oncorhynchus mykiss*. (**A**): Cortisol; (**B**): glycogen; (**C**): pyruvic acid; (**D**): ATP; (**E**): ADP; (**F**): AMP; (**G**): IMP; (**H**): Hx+HxR; (**I**): energy charge. Different uppercase letters mean significant differences (*p* < 0.05).

The cortisol and glycogen concentration were increased with ammonia stress and then decreased with ascorbic acid addition (*p* < 0.05) (Figure 5). In comparison with the control, the pyruvic acid content was severely decreased in the relief group and slightly decreased in the stress group. The ATP, AMP, and IMP content showed no significant difference among all conditions (*p* > 0.05). ADP was consumed in the treatment groups, but the Hx+HxR concentration was accumulated (*p* < 0.05). As for the energy charge, this was accelerated in the treatment groups (*p* < 0.05).

### 3.3. The Influence of Ascorbic Acid Addition on Muscle Flavor and Texture Under Ammonia Stress

**Figure 6:**
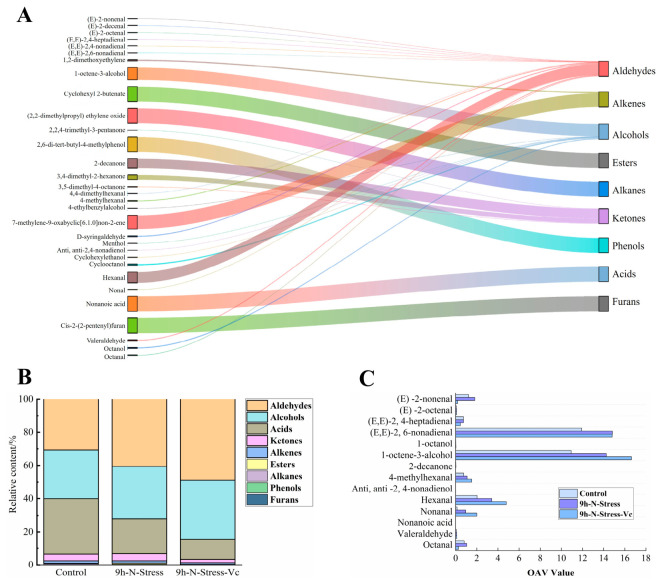
Effects of ascorbic acid addition on alleviating ammonia stress in muscle flavor in *Oncorhynchus mykiss*. (**A**): Sankey plot for the classification of major volatile organic compounds; (**B**): the relative contents of the main volatile organic compound categories in each group; (**C**): the OAV value of the main volatile organic compounds.

As shown in Figure 6A, a total of 28 volatile compounds were detected in all samples, including 13 aldehydes, 7 alcohols, 1 acid, 3 ketones, 2 alkenes, 1 ester, 1 alkane, 1 phenol, and 1 furan. Figure 6B presented the relative proportions of volatile compounds in each group. The relief group exhibited the largest total proportion of volatile compounds (23.07%), while the treatment groups showed the highest aldehyde content (30.58%). In the control group, acids accounted for the largest proportion (15.34%). The threshold of flavor compounds was calculated to assess the contribution of all detected volatile compounds to the overall flavor of the samples, and the result is presented in Figure 6C. The OAV values of 1-octen-3-ol, (E, E)-2, 6-nonadienal, hexanal, nonanal, and (E)-2-nonenal were the five highest values among all samples. All volatile compounds accumulated after ammonia stress; 1-octen-3-ol, 4-methylhexaldehyde, hexanal, and nonanal showed a severe increase in the relief group, and (E)-2-nonenal, (E, E)-2, 4-heptadienal, and octanal dropped.

**Figure 7:**
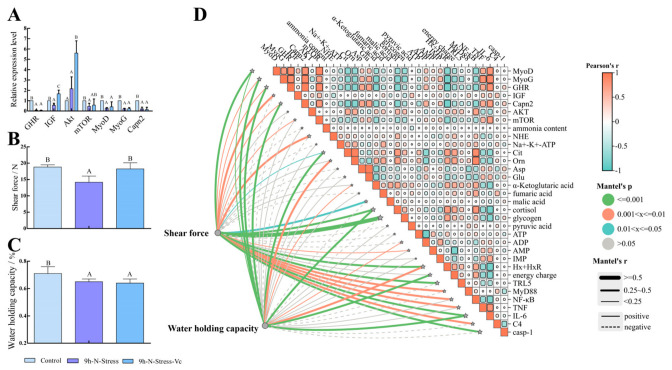
Effects of ascorbic acid addition on alleviating ammonia stress in muscle texture in *Oncorhynchus mykiss*. (**A**): The relative expression of genes (*GHR*, *IGF*, *Akt*, *mTOR*, *MyoD*, *MyoG*, and *Capn2*) related to muscle fiber differentiation; (**B**): shear force; (**C**): waterholding capacity; (**D**): Pearson’s correlation in muscle texture (shear force and water-holding capacity) and immune, apoptosis, ammonia metabolism, energy metabolism, and muscle fiber differentiation. Different uppercase letters mean significant differences (*p* < 0.05).

The results for the expression level of genes related to muscle fiber differentiation are presented in Figure 7A. All gene expression (*GHR*, *IGF*, *Akt*, *mTOR*, *MyoD*, *MyoG*, and *Capn2*) was decreased under stress (*p* < 0.05), except *Akt*. Ammonia addition slightly upregulated the mRNA expression levels of both *IGF* and *Akt*. Shear force reached its minimum at 9 h-ammonia stress (Figure 7B), while water holding capacity decreased at the same time point and remained low during recovery (Figure 7C).

## 4. Discussion

### 4.1. Ascorbic Acid Addition Relieved the Inflammatory Response Through TLR5/NF-κB Pathway, and Further Alleviated Apoptosis Occurrence and Damage to Immune Tissue Caused by Ammonia Stress

The result of ammonia levels in the blood, gill, kidney, and muscle suggested that the rainbow trout suffered from ammonia stress [11]. In this study, the ascorbic acid treatment significantly reduced blood ammonia content under ammonia stress (*p* < 0.05), suggesting partial alleviation of the systemic ammonia burden. However, ammonia concentrations in the gill and kidney remained statistically similar between the ammonia-exposed and ascorbic acid-treated groups (*p* > 0.05). This apparent inconsistency indicates that the effect of ascorbic acid on ammonia stress should not be interpreted as complete tissue ammonia clearance. The gill and kidney are directly involved in ammonia exchange, ion regulation, and nitrogen metabolism, and ammonia levels in these tissues may be influenced by the dynamic balance among ammonia uptake, transport, conversion, and excretion. Therefore, unchanged ammonia concentrations in the gill and kidney suggest that tissue ammonia accumulation was not fully reversed within the experimental period. Based on these results, ascorbic acid appears to mainly alleviate systemic ammonia burden, as reflected by reduced blood ammonia, and to modulate the genes related to ammonia handling and ion exchange rather than directly enhancing ammonia detoxification in all tissues. *NHE* is a key transporter mediating Na^+^/H^+^ exchange across the gill epithelium in fish [17]. Almost all the net inflow of Na^+^ outside and the net outflow of H^+^ inside fish are accomplished through the *NHE* in the gills. Active NH_4_^+^ was transported through the *NHE* and Na^+^-K^+^-ATPase [18]. In the present study, the *NHE* relative expression level increased, which indicated that ammonia stress modulated ion exchange and ammonia accumulation. However, the opposite trend was observed in the relief group, in which the *NHE* relative expression level decreased with ammonia accumulation in the gills and kidneys, which suggested that restricted H^+^ mediated ammonia elimination, caused a compensatory response to limit Na^+^ uptake, and generated a negative cell potential that caused NH_4_^+^ entry possibly via K^+^ channels [19]. This finding is consistent with the Na^+/^K^+^-ATPase activity reported by Sinha et al. [20] in a similar study on ammonia regulation in *Cyprinus carpio*.

The immune organ structure and function are of vital importance for the immune capacity of fish. Excessive ammonia levels caused by the necrosis of gill filaments and shedding of gill epithelial cells [21] result in gill function damage. As shown in Figure 2A, gill filaments showed congestion, epithelial tissue edema, epithelial cell necrosis, and shedding in the stress group, indicating that in rainbow trout under ammonia stress, the balance of the defense system was disrupted and in an emergency defense state. After ascorbic acid addition, gill filaments still showed layered fusion and vacuolation of epithelial cells, although these effects were substantially relieved compared to the stress group. After 12-h ammonia exposure, the gill tissue of four-finger threadfin juveniles exhibited physiological dysfunction and were in an emergency defense, which was alleviated by 48 h post-exposure [22]. Ascorbic acid in *Capoeta capoeta* exposed to dimethoate reduced gill lamella degeneration [23]. Prolonged ammonia exposure caused gill damage, leading to the accumulation of ammonia in the blood, which in turn triggered systemic physiological dysregulation and kidney injury via the circulatory system [24]. Ammonia accumulation caused kidney pathological damage, degeneration and even necrosis of tubular epithelial cells, and irregular cavities. Ammonia stress induced oxidative stress and an immunoinflammatory response, resulting in tissue damage in the gill, kidney, and muscle.

Immune organ injury consistently triggered changes in inflammatory-related gene expression. *TLR5* and *MyD88* were critical for initiating a rapid innate immune response: the upregulation of *TLR5* and *MyD88* expression indicated that ammonia stress activated the innate immune system and initiated downstream inflammatory response pathways. Although the expression patterns of inflammatory factors were not completely consistent among tissues, changes in *TLR5*, *MyD88*, *NF-κB*, and downstream inflammatory genes suggested the common involvement of the *TLR5/MyD88/NF-κB*-related inflammatory axis under ammonia stress. Therefore, the pathway proposed in this study should be interpreted as a commonly involved inflammatory response framework rather than an identical regulatory pattern across all organs. The tissue-dependent differences in inflammatory gene expression may reflect variations in local inflammatory intensity, stress sensitivity, and feedback regulation. However, the present study did not further dissect the organ-specific regulation of this pathway, which represents a limitation. Lin et al. [25] demonstrated that waterborne microcystin-LR induced persistent inflammation through the *TLR/MyD88* signaling pathway. *NF-κB/TNF/IL-6* were direct indicators of the intensity of the inflammatory response. The upregulation of NF-κB/TNF/IL-6 expression indicated an inflammatory response, which likely contributed to tissue damage (Figure 2) and apoptosis occurrence (Figure 3). This inflammatory state may have further disrupted systemic physiological homeostasis [26], leading to elevated energy expenditure, growth inhibition, and finally death. It has been reported that transcriptional and translational levels of genes involved in the *TLR 5/NF-κB* pathway including *TLR 5*, *NF-κB*, *IL-1β*, and *TNFα* were increased, which was caused by intestinal inflammation strongly induced by carbendazim (0.2–20 µg/L) in *Ctenopharyngodon idella* [27]. The expression changes of *C4* reflected that the effect mechanism of innate immunity was synergistically activated when the *TLR5* signaling pathway was initiated. The downregulation of *C4* in fish under ammonia stress implied that the ammonia inhibited innate immunity activation, and ascorbic acid addition upregulated *C4* and synergistically activated innate immunity. Similarly, ammonia and nitrite activated immunity through increasing the level of *C3* and *C4* in the kidneys in *Hypophthalmichthys molitrix* [28]. *Casp-1* functioned as the key executioner of pyroptotic cell death. Although the expression of *Casp-1* had no significance in gill and muscle, and was significantly downregulated in the ammonia and relief groups, the TUNEL analysis showed that the ammonia stress induced the occurrence of apoptotic cell death, and ascorbic acid addition effectively reduced apoptosis occurrence. Ascorbic acid addition exhibited remarkably decline in ROS production, apoptotic cell ratio and DNA damage in *Takifugu obscurus* following the exposure to low temperature stress [29]. Ascorbic acid inhibited the increase in metabolism enzymes and inflammatory factors and activated immune and antioxidant signaling pathways to relieve oxidant stress, immune response, and apoptosis in live tiger grouper during simulated transport [30]. These results demonstrated that ascorbic acid addition can relieve the inflammatory response through changes in mRNA expression of genes involved in the *TLR5/MyD88/NF-κB* pathway, further alleviating apoptosis occurrence and immune tissue damage caused by ammonia stress.

### 4.2. Ascorbic Acid Addition Affected Energy Metabolism, and Sped Up Ammonia Ion-Exchange Responses Through Ammonia Transport and Metabolism

To prevent excess endogenous ammonia accumulation and further immune damage, the fish tended to efficiently excrete or detoxify ammonia to alleviate these negative effects [31]. There are two primary ammonia detoxification pathways, the glutamine synthesis pathway and the ornithine cycle pathway [32], which exhibit a trend of synergistic enhancement relation between them, the former one for rapid and acute detoxification and the latter one for chronic ammonia, with both working in concert to mitigate ammonia toxicity in the rainbow trout [33]. Glutamine synthetase is a key rate-limiting enzyme, which catalyzes the synthesis of glutamine from glutamate and ammonia [34]. In the present study, decreased glutamate levels and upregulated *GS*-related gene expression were observed in the ammonia-stressed and ascorbic acid-treated groups. These changes may indicate the potential involvement of the glutamate–glutamine pathway in ammonia stress adaptation. However, because glutamine concentrations were not directly measured, this interpretation should be considered indirect evidence of increased glutamine synthesis capacity rather than direct confirmation of enhanced glutamine production. This method of ammonia detoxification has been reported in *Paramisgurnus dabryanus* [35] and *Eriocheir sinensis* [36]. Glutamine can be stored in tissues and then used as an oxidizing substrate once back to normal, but the drawback is the energy cost of ammonia detoxification [37]. At the same time, glutamate is primarily produced by the reductive amination of α-ketoglutarate, an intermediate product of the TCA cycle [38], so a well-functioning TCA cycle is necessary for ammonia detoxification [39], not only for the energy supply but also to provide carbon skeletons for the ornithine cycle, another strategy for ammonia detoxification [40,41]. Arginine, a crucial substrate of the ornithine cycle pathway, is hydrolyzed to ornithine and urea. Ornithine progresses through the cycle by reacting with carbamoyl phosphate to form citrulline, and then citrulline combined with aspartic acid again is converted to succinate and arginine, and finally the cycle restarts [42]. In the investigation, arginine was diminished after stress and further declined with ascorbic acid, and succinate was increased in the ammonia stress and relief groups, which suggested that ammonia stress and ascorbic acid addition affected the cycle. Ornithine and citrulline levels were elevated in both the stress and relief groups. In contrast, aspartic acid decreased, likely due to its consumption in the subsequent urea cycle for citrulline-aspartate condensation. Fumarate and malate serve as carbon skeletons for oxaloacetate, which is subsequently converted to aspartic acid via transamination [43]. Only a significant fumarate increase in the relief group was observed in our study. These results demonstrated that the strategy against ammonia stress was the ornithine cycle that converts highly toxic NH_3_ into non-toxic urea, which was accelerated with ascorbic acid addition for faster detoxification. However, the ornithine cycle is considered inefficient in rainbow trout, with only 10–15% of nitrogenous waste excreted [44]. Ascorbic acid supplementation was associated with alterations in ammonia-related metabolism, primarily involving *GS*-mediated glutamate–glutamine conversion, with the ornithine cycle playing a secondary role in nitrogen redistribution under ammonia stress. Hence, although the ion-exchange response was sped up by ascorbic acid addition, the inflammatory response, apoptosis occurrence, and immune tissue damage occurred inevitably but was alleviated compared with ammonia stress (as shown in Section 4.1).

Energy metabolism is the major pathway used to cope with ammonia exposure [40]. To counteract ammonia stress, the content of cortisol, regarded as a key signaling molecule to mobilize energy reserves, rose in the rainbow trout [45]. Usually, cortisol prompts glycogenolysis and gluconeogenesis; the former is involved in the acute and rapid stress response and the latter is involved in the chronic stress response [46]. As ammonia stress was maintained for 9 h, gluconeogenesis was on the rise, which led to an increase in glycogen content and a decrease in pyruvic acid content in the ammonia and relief groups. The differences in physiological responses between the stress and relief groups were likely attributable to ascorbic acid addition to alleviate ammonia toxicity through enhanced ammonia detoxification and ion-regulatory capacity. Consequently, the reduced energy demand in the relief group was reflected by lower levels of cortisol, glycogen, and pyruvic acid than those observed under ammonia stress. Similar results have been reported for the effect of stocking density on glucose metabolism in hybrid sturgeon [47]. Exposed to stress, this fish generates and rapidly consumes an amount of ATP to counteract stress to maintain its basic function [48], which might have led to the lack of a significant difference among all groups because of the fast consumption of ATP. The ADP content was decreased in the stress and relief groups, which was caused by ATP and its derivatives degrading rapidly. Then, the accumulation of Hx and HxR (end products of ATP catabolism) was accompanied by an increase in energy charge in both the ammonia-stressed and relief groups. Overall, we concluded that ascorbic acid addition affected energy metabolism, and modulated ammonia handling and ion-exchange responses through ammonia transport and metabolism.

### 4.3. Ascorbic Acid Addition Reduced Muscle Flavor and Texture Deterioration Caused by Ammonia Stress

As mentioned above, the fiber gaps became larger and irregular (Figure 2C) and the Hx+HxR content accumulated (Figure 5H) after ammonia exposure, and the addition of ascorbic acid mitigated these gaps and the Hx+HxR accumulation, which suggested that ascorbic acid had a positive effect on the muscle texture and flavor in rainbow trout exposed to ammonia stress. As reported by Shi et al. [49], *Micropterus salmoides* under chronic alkalinity stress showed a significant enhancement of polyunsaturated fatty acid and free acid at 14 mmol/L and showed an extreme improvement in hardness and chewiness at 28 mmol/L. Furthermore, *Larimichthys crocea* were reared at a higher stocking density (7.01 kg/m^3^) exhibited more umami and salty taste, along with superior water holding capacity, compared with fish at normal density (4.01 kg/m^3^) [50]. However, the specific changes in rainbow trout flavor and texture under ammonia stress and ascorbic acid relief have not been clarified yet. Hence, volatile organic compounds, the expression of genes related to muscle fiber synthesis and degradation, shear force, water-holding capacity, and the Pearson correlation were analyzed to explain this.

GC-MS effectively separated the signal peaks of volatile organic compounds, finding a total of 31 volatile organic compounds (Figure 6A). Aldehydes, mainly originated from the oxidation and degradation of fatty acids, was the highest in the detection of volatile components. The unsaturated fatty acid content in rainbow trout decreases after fishing stress [51], which might be attacked by reactive oxygen species and then oxidized and degraded into aldehydes; therefore, the aldehyde content was increased after ammonia exposure in our study. Hexanal, nonanal, and octanal are key volatile biomarkers of lipid oxidation in fish [52]. Hexanal is regarded as a general indicator of lipid oxidation, while nonanal is the oxidation degradation product of oleic acid, and octanal primarily originates from ω-3 (e.g., eicosapentaenoic acid) and ω-7 (e.g., palmitoleic acid) unsaturated fatty acids. Moreover, (E)-2-nonenal is typically generated by the oxidation of linoleic acid and arachidonic acid, and (E, E)-2, 4-heptadienal is regarded as the oxidation product of alpha-linolenic acid and EPA. They are the main contributors to the earthy–moldy, ichthyophiid, and herbaceous flavors of aldehydes, which are considered the main flavor compounds responsible for off-flavors in fish and fish products [53]. These aldehyde flavor substances were increased after ammonia stress and reduced with ascorbic acid addition, except for hexanal and nonanal, which had a higher content in the relief group. And the high content of (E, E)-2, 6-nonadienal further intensified the production of discordant flavors among all groups. Alcohols are primarily generated through the decomposition of fatty acid hydroperoxides, as well as via the enzymatic activity of lipoxygenases and the reduction of carbonyl compounds [54]. 1-octene-3-alcohol is regarded as having a fishy and mushroom-like aroma [55] and is one of the causes of the earthy taste of freshwater fish [56]. The content of 1-octene-3-alcohol accumulated with ammonia exposure and showed a higher increase in the relief group. Therefore, ascorbic acid addition could slow down unsaturated fatty acid oxidation caused by ammonia stress to reduce earthy–moldy and fishy off-flavors in muscle, but cannot completely counteract the off-flavors. Moreover, ascorbic acid altered the volatile compound profile under ammonia stress, while its actual sensory effect requires further validation.

The *GHR-IGF-Akt-mTOR* signaling pathway regulates cell growth, proliferation, and metabolism [57]. In this study, the relative expression of *GHR*, *IGF*, and *mTOR* were downregulated with ammonia stress, and an increase in *Akt* expression was also observed but did not reach statistical significance, while *IGF*, *Akt*, and *mTOR* mRNA expression were increased in the relief group. *MyoD* and *MyoG* are crucial genes for myogenesis and the keys to initiate the myogenic program and drive the terminal differentiation of myoblasts, respectively [55]. *MyoD* and *MyoG* gene expression were decreased, and *Capn 2* was also reduced in the ammonia group and relief group. *Capn 2* induces the degradation of myofibrillar structural proteins, thereby playing a critical role in cellular responses to environmental stress [58]. The results demonstrated that ammonia stress had a negative effect on the partial modulation of muscle growth and differentiation-related gene expression, reduced potential involvement in maintaining myofibrillar integrity, and finally may have led to a decrease in the water-holding capacity and the shear force (Figure 7B,C). Dietary L-theanine supplementation increased the expression of *CAPN1*, *CAPN2*, and integrin *β1* in the gastrocnemius muscle of diquat-stressed mice and decreased muscle drip loss [59].

Based on the Pearson correlation, the correlation network revealed that the changes in muscle texture (shear force and water-holding capacity) were significantly linked to immunity, apoptosis, ammonia metabolism, energy metabolism, and muscle fiber differentiation (Figure 7D), which suggested that ammonia stress-induced changes in the cortisol response, ammonia handling, energy metabolism, inflammatory/apoptotic signaling, and muscle fiber differentiation or degradation may be linked to the loosening of muscle structure and reductions in shear force and water-holding capacity. However, due to the absence of an ascorbic acid-only group, these effects should be interpreted as protective responses under ammonia exposure rather than as independent physiological effects of ascorbic acid.

## 5. Conclusions

In conclusion, ammonia stress caused inflammatory responses, apoptosis, tissue damage, and ammonia intoxication in rainbow trout, resulting in the deterioration of muscle texture and flavor quality. Ascorbic acid alleviated these adverse effects by modulating ion exchange and regulating the mRNA expression of genes involved in the *TLR5/MyD88/NF-κB* pathway, thereby suppressing inflammation and apoptosis occurrence. At the same time, ascorbic acid promoted glutamate utilization and activated the ornithine cycle, which facilitated ammonia detoxification and coordinated energy metabolism under ammonia stress. In addition, ascorbic acid improved myofibrillar structure and shear force, and reduced the accumulation of key off-flavor compounds such as 1-octen-3-ol and (E)-2-nonenal. These findings suggest that ascorbic acid is a feasible strategy for mitigating ammonia-induced muscle quality deterioration in rainbow trout.

## Data Availability

The original contributions presented in this study are included in the article/Supplementary Materials. Further inquiries can be directed to the corresponding authors.

## Supplementary Materials

The following supporting information can be downloaded at: https://www.mdpi.com/article/10.3390/foods15132316/s1, Table S1: Oligonucleotide primers used in the reverse transcription-qPCR analysis; Supplementary Material S2: Quantitative PCR; Supplementary Material S3: Organic acid determination; Supplementary Material S4: Volatile compounds determination.
