# Supplementary material for: Effects of Ascorbic Acid on Apoptosis, Metabolism, and Muscle Quality in Ammonia-Stressed Rainbow Trout (Oncorhynchus mykiss)
Source: Foods. 2026 Jun 30;15(13):2316. doi: 10.3390/foods15132316 (PMC13361369; doi:10.3390/foods15132316)
Supplement: Supplementary file 1 [file foods-15-02316-s001.zip › foods-4389287-supplementary.pdf]

Supplementary material

Effects of Ascorbic Acid on Apoptosis, Metabolism, and Muscle Quality in Ammonia-Stressed Rainbow Trout (*Oncorhynchus mykiss*)

Siliang Yuan [1,†], Yiwen Wu [2,3,†], Yuxuan Pi [2,4], Chenxin Wang [2,5], Guangquan Xiong [2], Wenjin Wu [2], Liu Shi [2], Tao Yin [3], Hao Du [1], Lan Wang [2,*] and Sheng Chen [2,*]

[1] Yangtze River Fisheries Research Institute, Chinese Academy of Fishery Sciences, Wuhan 430223, China
[2] Key Laboratory of Agricultural Products Cold Chain Logistics, Ministry of Agriculture and Rural Affairs, Hubei Key Laboratory of Characteristic Resources and Utilization, Institute of Agro-Products Processing and Nuclear Agricultural Technology, Hubei Academy of Agricultural Sciences, Wuhan 430064, China
[3] College of Food Science and Technology, Huazhong Agricultural University, Wuhan 430070, China
[4] School of Bioengineering and Food Science, Hubei University of Technology, Wuhan 430068, China
[5] College of Biological and Food Engineering, Hubei Minzu University, Enshi 445000, China
* Correspondence: lilywang_2016@163.com (L.W.); cs55490964@163.com (S.C.)
† These authors contributed equally to this work.

Supplementary Material S1.

Table S1. Oligonucleotide primers used in the reverse transcription-qPCR analysis

Gene	Primer Sequence 5′-3′
*β-actin*_F	TCAACCCCAAAGCCAACAGG
*β-actin*_R	TACCGCAAGACTCCATACCGAG
*NHE*_F	ATGACATTGTGGTGGAGGTGG
*NHE*_R	TGGCTTTGAAGTGGTTGTAGGC
*TLR 5*_F	CACTGCCTCCATACATTACCCA
*TLR 5*_R	AAGCCAAGCAAATCTTCCTCAC
*MyD88*_F	CCCGAGAAACACTGTGGCAT
*MyD88*_R	GCCACAGTGTTTCTCGGGTTTA
*NF-κB*_F	TAGGTTATGGGAGCCGAGGAT
*NF-κB*_R	TGAGATATGGGGATGGGTTGTG
*TNF*_F	ACCTCTCCTCGGGTTCCTCATT
*TNF*_R	TCTCACACTCCCCAAGTCTCCT
*IL-6*_F	CAATCAACCCTACTCCCCTCTG
*IL-6*_R	AAGTCTTTGCCCCTCTTTCCC
*C4*_F	TGGGGTGACAGGCAGAAAGA
*C4*_R	AAAGCTCGGCAGCACAAATT
*casp-1*_F	ACACCATCATCGCCCCAGTT
*casp-1*_R	CCACATCATATCCCAGATCCCT
*GS*_F	AGAAGGGCTACTTTGAGGACCG
*GS*_R	AACAACCAACACGAGAGGGGAAAGA
*GHR*_F	GGAAAGGAGAAAGAGAAGCCG
*GHR*_R	AGTGAGGAAACGGGCGAGGCAGGTG
*IGF*_F	AGTGCGATGTGCTGTGTCTCCT
*IGF*_R	TGTGCCTCTGTCCACGTTTTG
*Akt*_F	ACGGTGAGGAACAGAGTGGAGA
*Akt*_R	AAGTGGGTGGAAGACTGGGATA
*mTOR*_F	CCAGCCTCTCTACGCAAAAACC
*mTOR*_R	CAACCTCAAAGCAATCCCCAA
MyoD _F	CCACCAAGACGGAGAAACAAGT
MyoD _R	AACCATCCCCGGGAGAACAT
MyoG _F	AACCCCTACTTCTTCCCCGA
MyoG _R	GTTGCCTTGTCCTCCATACCTC
*Capn2*_F	TGCTAAGCGAACGGACATAAAG
*Capn2*_R	TACCGAGCTGCCAAAACCTG

Supplementary Material S2. Quantitative PCR

Total RNA was extracted from tissue (six biologically independent samples per group) using trizol reagent (R701, Vazyme Biotech Co.,Ltd, Nanjing, China). First-strand cDNA synthesis was acquired through commercial kit (R323) (Vazyme Biotech Co.,Ltd, Nanjing, China). cDNA purity was tested with NanoDrop 2000 (Thermo Fisher Scientific, USA). The 10 μL reaction system consisted of 5 μL of master Mix (Vazyme, Nanjing, China), 0.5 μL of each primer mix (0.2 μmol/L), 1 μL of cDNA and 3.5 μL of nuclease-free ddH_2O was added in PIKOREAL 96 PCR System (Thermo Fisher Scientific, USA). The PCR thermal cycling program was as follows: 95 °C for 5 min, followed by 40 cycles of 95 °C for 10 s and 60 °C for 30 s.

Supplementary Material S3. Organic acid determination

The tissue (0.5 g) was weighed and homogenized with 20 mL ddH_2O at 4000 rpm for 1 min, then centrifugated for 10 min (4 000 rpm, 4 ^{0}C). The supernatant was collected and the precipitate was diluted by another 20 mL ddH_2O followed with being centrifugated for 10 min (4 000 rpm, 4 ^{0}C). All supernatant was collected and filled to 50 mL, then filtered through a 0.45 μm, finally separated by high performance liquid chromatography (Ultimate 3000, Thermo Fisher Scientific, Germany). The fractions were separated in 0.8 mL/min, 0.1% phosphate- methanol and detected at 210 nm SPD-10A (V) detector.

Supplementary Material S4. Volatile compounds determination

Samples (0.1 g freeze-dried sample,) were weighed, transferred to 20 mL headspace vials, and added 20 μL 2-Methyl-3-heptanone (0.816 μg/mL), 1.5 g NaCl and 5 mL ddH_2O. Sample incubation was conducted at 60 °C for 30 min with an

agitator speed of 500 rpm. The volatile substances were separated through a VF-WAX capillary column (25 m × 0.25 mm × 0.2 μm, CP9024, Agilent Technologies Co., Ltd., Palo Alto, CA, USA). analyzed by gas chromatography (8890, Agilent Technologies Co., Ltd., Palo Alto, CA, USA) equipped with mass selective detector (7000D, Agilent Technologies Co. Ltd., Palo Alto, CA, USA). The MS was operated under electron impact (EI) mode at 70 eV. The ion source temperature was 230 °C, and the temperature of the quadrupole was set at 150 °C. The temperature program started at 40 °C, increased to 100 °C at 5 °C/min, then to 230 °C at 15 °C/min, held for 5 min, followed by 230 °C for 2 min, and maintained for an additional 5 min. The data were collected at a rate of 3.2 scan/s over a range of m/z 30–1000.
